# Normal and Pathological Tau Uptake Mediated by M1/M3 Muscarinic Receptors Promotes Opposite Neuronal Changes

**DOI:** 10.3389/fncel.2019.00403

**Published:** 2019-09-04

**Authors:** Viktoriya Morozova, Leah S. Cohen, Ali El-Hadi Makki, Alison Shur, Guillermo Pilar, Abdeslem El Idrissi, Alejandra D. Alonso

**Affiliations:** ^1^Department of Biology and Center for Developmental Neuroscience, College of Staten Island, The City University of New York, Staten Island, NY, United States; ^2^Biology Program, The Graduate Center, The City University of New York, New York, NY, United States; ^3^Department of Neurosciences, Case Western Reserve University, Cleveland, OH, United States

**Keywords:** tau, muscarinic receptors, uptake, neurodegeneration, Alzheimer’s disease

## Abstract

The microtubule associated protein tau is mainly found in the cell’s cytosol but recently it was also shown in the extracellular space. In neurodegenerative diseases, like Alzheimer’s disease (AD), pathological tau spreads from neuron to neuron enhancing neurodegeneration. Here, we show that HEK293 cells and neurons in culture uptake extracellular normal and pathological Tau. Muscarinic receptor antagonists atropine and pirenzepine block 80% this uptake. CHO cells do not express these receptors therefore cannot uptake tau, unless transfected with M1 and/or M3 receptor. These results strongly suggest that muscarinic receptors mediate this process. Uptake of normal tau in neurons enhances neuronal process formation but a pseudophosphorylated form of tau (pathological human tau, PH-Tau) disrupts them and accumulates in the somatodendritic compartment. AD hyperphosphorylated tau (AD P-Tau) has similar effects as PH-Tau on cultured neurons. Addition of either PH-Tau or AD P-tau to neuronal cultures induced microglial activation. In conclusion, uptake of extracellular tau is mediated by muscarinic receptors with opposite effects: normal tau stabilizes neurites; whereas pathological tau disrupts this process leading to neurodegeneration.

## Introduction

Tau is a neuronal cytosolic microtubule associated phospho-protein whose function can be influenced by post-translational modifications. Abnormally phosphorylated tau is a common denominator in several types of dementia, including Alzheimer’s disease (AD) and other tauopathies (Goedert et al., 2010; Beharry et al., 2014).

Unlike normal tau, soluble hyperphosphorylated tau taken from AD patients (AD P-tau) does not promote microtubule assembly. Instead, AD P-tau binds normal tau, sequestering it from interactions with tubulin and producing bundles of filaments observed by electron microscopy (Alonso et al., 1996). This suggests that differences in tau phosphorylation states could affect its conformation and subsequently alter protein-protein interactions and tau biological function. Seeding activity into self-assembled aggregates can lead to the development of neurofibrillary tangles. We have shown previously that pseudophosphorylation at Ser199, Thr212, Thr231, and Ser262 with inclusion of the FTDP-17 R406W mutation (Pathological Human Tau, PH-Tau) results in a protein that mimics the effect of AD P-tau in culture (Alonso et al., 2010). This protein has been studied in mammalian cell culture, Drosophila, and mouse models. The expression of PH-Tau induced microtubule disruption in all systems, and resulted in cognitive and behavior impairment in Drosophila and mice (Alonso et al., 2010; Beharry et al., 2013; Di et al., 2016).

In AD, the initial neurodegenerative lesions are in the transentorhinal cortex spreading slowly to the hippocampus and neocortex (Hyman et al., 1984; Braak and Braak, 1991; Goedert et al., 2010), leading to cognitive decline. It was proposed that pathological tau is spread from cell to cell in a prion-like fashion (Holmes and Diamond, 2014; Mudher et al., 2017). The seeding and propagation of filamentous structures by hyperpshosphorylated tau has been a central point in AD research (Alonso et al., 2016; Hu et al., 2016; Iqbal et al., 2016; Mudher et al., 2017; Shafiei et al., 2017). Cells in culture take up tau aggregates ranging in size from oligomers and short filaments (neuronal cells) to fibrils (non-neuronal cells) through endocytosis (Frost et al., 2009; Nonaka et al., 2010; Guo and Lee, 2011; Wu et al., 2013; Sanders et al., 2014; Wu et al., 2016). Recently, it has been shown that monomeric tau can enter neurons (Evans et al., 2018) and based on the kinetics of tau uptake, it was suggested that this might be a receptor-mediated process (Evans et al., 2018). Furthermore, intracerebral injections of abnormal tau showed that tau pathology propagates away from the injection site, to neighboring brain regions indicating that tau can be transferred from cell to cell (Bolmont et al., 2007; Clavaguera et al., 2009; Lasagna-Reeves et al., 2012; Clavaguera et al., 2013; Iba et al., 2013; Wu et al., 2013; Ahmed et al., 2014; Clavaguera et al., 2014). Taken together, these results have led to the tau propagation hypothesis in which tau is released from one neuron into the intra-neuronal space, and taken up by other neurons transferring tau toxicity in a similar fashion as prion proteins (as reviewed in Takeda, 2019).

In this study we demonstrate that addition of recombinant tau protein to the culture media of Human Embryonic Kidney cells (HEK293) and primary cerebellar neuronal culture results in the uptake of tau and PH-Tau. Chinese Hamster Ovary (CHO) cells are unable to uptake tau unless they are transfected with M1- and M3-type muscarinic receptors. Furthermore, uptake into both HEK293 cells and cerebellar neurons is blocked by atropine, a muscarinic receptor antagonist, and uptake in cerebellar neurons is blocked by pirenzepine, an M1 antagonist, but not AF-DX116, an M2 antagonist, or pertussis toxin (PTX), an M2/M4 antagonist. Neuronal cultures incubated with wild-type tau for 7 days show an abundant and well-organized neuritic arbor. Similar cultures exposed to PH-Tau and AD P-tau show accumulation of tau in the somatodendritic neuronal compartment and disruption of neuronal processes. Our experimental results suggest that both tau and pathogenic tau are taken up in mammalian cells and neurons in a muscarinic receptor-mediated uptake mechanism that facilitates long-term neuronal changes.

## Materials and Methods

### Antibodies

The primary antibodies used in this work are as follows: tau-13 (mouse, 1:1,000,000 for Western blot, 1:1000 for immunohistochemistry), CHRM1 (rabbit, 1:1000, Invitrogen, Cat#711098), βactin (1:2500, Santa Cruz, Cat#SC4778), β-III tubulin (chicken, 1:1000, Abcam, Cat#AB107216), GFAP (rabbit, 1:1000, Invitrogen Cat#PA3-16-727), IbaI (rabbit, 1:1000, Wako Cat#019-19741). The secondary antibodies used in this work are as follows: anti-mouse IgG-HRP (1:2000, Santa Cruz, Cat#SC-516102), anti-mouse Alexa 488 (1:1000, Invitrogen, Cat#A21202), anti-chicken Alexa 555 (1:1000, Invitrogen, Cat#A21437), anti-rabbit Alexa 594 (1:1000, Invitrogen, Cat#A21203). Tau-13 antibodies were a gift from Dr. Nicholas Kanaan at Michigan State University (originally created by Dr. Lester Binder at Northwestern University).

### Recombinant Protein Expression and Purification

GST-tagged proteins were transformed into BL21(DE3)pLysS cells and purified using glutathione-sepharose beads (GE Healthcare) as described (Cevher et al., 2010).

### Protein Quantitation, PAGE, and Western Blot Analysis

Quantitation of protein samples was performed by either the Bradford assay or by UV quantitation. The Bradford assay was performed on recombinant proteins and cell and neuronal lysates as described by the BioRad manual. Recombinant protein was also determined by UV spectroscopy at 280 nm using the extinction coefficients of 50685 M^–1^cm^–1^ for GST-tau, 56185 M^–1^cm^–1^ for GST-PH-Tau, and 43110 M^–1^cm^–1^ for GST. Recombinant proteins (10 μg per lane), cell and neuronal lysates (7 μg per lane) were analyzed by SDS-PAGE and Western blot as described with some modifications (Grundke-Iqbal et al., 1984).

### Cell Culture and Transfection

HEK293 cells were grown in DMEM cell media supplemented with 10% Fetal Bovine Serum (FBS), sodium pyruvate, Glutamax, and Antibiotic-Antimitotic. CHO cells were grown in FK12 media with 10% FBS, sodium pyruvate, Glutamax and Antibiotic-Antimitotic. Media components were from Gibco or Thermo Fisher Scientific.

Chinese Hamster Ovary cells were plated on glass coverslips overnight. Transfections were performed using LipofectamineTM 2000 (Invitrogen, Carlsbad, CA, United States), according to the manufacturer’s instructions. Briefly, transfections were performed in serum-free media OptiMEM using 3 μg of total DNA and 3 μl of Lipofectamine^TM^ 2000 per 35 mm dish. Cells were incubated in transfection media for 4 h and then replaced with fresh cell media.

### Preparation of Mixed Cerebellar Neuronal Culture

Cerebellar granule cells were prepared from PN7 CD1 mice. The cerebellum was removed and single cell suspension was prepared by trypsinization and trituration in 1% trypsin in Ca^2+^, Mg^2+^ free isotonic phosphate buffer (CMF-PBS). Cells were washed in CMF-PBS and resuspended in minimum essential culture medium supplemented with 0.25% glucose, 2 mM GlutaMax, 10% Horse Serum, 5% FBS, and 25 U/ml both penicillin and streptomycin. Cells were seeded into PDL-coated dishes and incubated at 37°C, 5% CO_2_. Twenty-four hours after neuronal isolation, culture media was changed to media containing 15% of N2 supplement (R&S system) and 10 μM KCl. This media was changed every 2 days. Media components were from Gibco, Sigma, or Corning.

### Protein Addition to Cultures

GST, GST-tau, and GST-PH-Tau (0.4 μg/ml final concentration) were added to the cell media of HEK293 cells, CHO cells and primary neuronal cultures. HEK293 and CHO cells were incubated with proteins for 2–4 h and washed with PBS. After additional incubation for 48 h, cells were fixed, processed for immunocytochemistry and viewed by confocal microscopy. M1/M3 transfected CHO cells were incubated for 48 h, then tau or PH-tau was added to the cells as described, incubated for 4 h, then media was changed and cells were grown for another 24 h and then processed for western blot.

Neuronal cultures were incubated for 24 h in the presence of tau or PH-Tau and were either fixed and processed for immunochemistry (DIV8) or incubated for longer time periods. For longer incubation times, half of the media was removed after 24 h and replaced with fresh media. The cultures were fixed and processed at DIV10, DIV12, or DIV14.

### Blocking of Muscarinic Receptors

HEK293 cells and neuronal cultures were treated with 100 μM Atropine sulfate salt monohydrate (Sigma), 10 μM Pirenzepine Dichloride (Alfa Aesar), 40 ng/ml Pertussis toxin (Enzo), or 10 μM AF-DX116 (Toriks Biochemicals) in the culture media for 10 min prior to protein addition. After pre-treatment, half of the media was removed, replaced with fresh media and tau, PH-Tau or AD P-tau were added immediately to the cultures and incubated as described above.

### Immunocytochemistry of Cell and Neuronal Cultures

HEK293 and CHO cells were fixed with cold methanol at −20°C for 5 min. Fixed cells were washed with PBS three times for 5 min and then blocked with 5% Donkey serum in PBS for 1 h at room temperature. Tau-13 was added to blocking buffer and cells were incubated overnight at 4°C with agitation. Cells were washed three times for 5 min each and incubated with anti-mouse Alexa 488 in blocking buffer (1:1000 dilution) for 2 h. Finally, cells were washed three times with PBS and the coverslips were mounted on the slides using Vectashield mounting media containing DAPI (Vector Laboratories, Inc.) and viewed by confocal microscopy.

Neuronal cultures were fixed with warm 4% paraformaldehyde for 10 min at room temperature, washed with PBS three times for 5 min and permeabilized with 100% methanol at −20°C for 10 min. Permeabilized cells were washed again with PBS three times for 5 min and blocked with 5% BSA, 1% Donkey serum, 0.2% TritonX-100 in PBS for 1 h at room temperature. Tau-13, β-III tubulin, GFAP, or IbaI were added to blocking buffer and cultures were incubated overnight at 4°C. Cultures were washed with 0.2% TritonX-100 in PBS (PBS-T) three times 5 min each and incubated with anti-mouse Alexa 488, anti-chicken Alexa 555, or anti-rabbit Alexa 594 in blocking buffer for 2 h at room temperature. Cultures were washed with PBS-T and then with PBS. Coverslips were mounted as described above.

### LIVE/DEAD Imaging

Neuronal cultures (DIV 14) cultured in 24-well plates were treated with reagents from the LIVE/DEAD Cell Imaging Kit (488/570) (Invitrogen) as described by the manufacturer’s protocol. Analysis was performed using a Zeiss-Axio Observer Z1 Live Imager.

### Stereotaxic Injections

Five-month-old CD-1 mice were anesthetized with 100 mg/kg ketamine and mounted onto a stereotaxic apparatus, and kept anesthetized under isofluorane throughout the procedure. A midsagittal incision was made and a hole was drilled using a Circuit Medic Micro Drill System into the cranium according to the following coordinates set from bregma: AP: + 2.18 mm, ML: + 2.85 mm, DV: −2.5 mm. The mice were injected with 2 μL of 2 μg/mL of either aCSF, tau, or PH-Tau with the use of a Hamilton syringe at a rate of 0.5 μL/min for 4 min of total injection time with a KD Scientific Syringe Infusion Pump. The incision was treated with garamycin and closed with surgical staples, and the mice were placed onto a heating pad while they recovered from the procedure. The mice were sacrificed after 45 days.

### Cryosectioning and H&E Staining

Mice were anesthetized with 0.3 mL urethane (1 g/mL) and transcardially perfused with 0.1 M phosphate buffered saline (PBS) and 4% paraformaldehyde (PFA) in 0.1 M PBS then stored in PFA followed by 30% sucrose at 4°C. Brains were cryosectioned in sagittal vibratome sections (50 μm) and mounted onto microscope slides. Tissue sections were then incubated in the following order: PBS for 5 min, dH_2_O for 1 min, hematoxylin for 4 min, running tap water for 3–5 min, dH_2_O for 1 min, eosin for 2 min, 70% alcohol for 2 min, 95% alcohol for 2 min, 100% alcohol for 2 min, and xylene for 2 min. The tissue sections were covered in a Permount/xylene solution and coverslipped. The staining was observed with an EVOS PLc light microscope at 20×.

### Confocal Image Acquisition, Measurements, and Statistical Analysis

Images for each set of analysis were observed under a LEICA TGS SP5 confocal laser scaning microscope (Leica Microsystems CMSGmbH, Mannheim, Germany) and obtained using the same gain, laser intensity and pinhole size. Confocal images were analyzed using ImageJ software. Images were split onto blue (DAPI), green and red channels. The same threshold was assigned to all pictures for each channel. Mean gray value was calculated from each channel and divided by the number of the nuclei. The results were graphed and compared using Excel 2010.

All experiments were performed at least three times and at least 10 images from each experiment were analyzed. Statistics were performed using Microsoft Excel 2010. Two-Sample *t*-Test assuming equal variances was used to compare samples. The data reported as mean values. An alpha level of 0.05 was used for all statistical tests. The significance was determined based on two-tailed *P* values.

## Results

### Tau Uptake Into Cells Is Receptor Mediated

**FIGURE 1:**
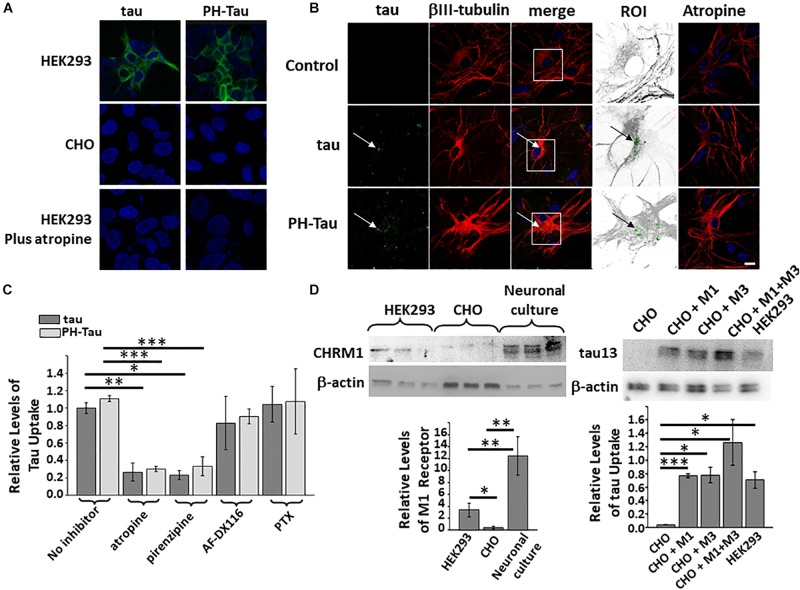
Uptake of tau is cell-type specific. Recombinant tau and PH-Tau (400 ng/mL) were added to HEK293 and CHO cells **(A)** and to primary neuronal cultures **(B)**. Confocal analysis indicated that uptake occurred in HEK293 and neuronal cultures (white arrows) but not CHO cells and was blocked by pre-treatment with atropine. In the neuronal images, the boxed areas were zoomed 2.5× and converted (βIII-tubulin, gray; tau, green). **(C)** Quantitation of tau uptake into neuronal cultures indicates that both tau (dark bars) and PH-Tau (light bars) are uptaken at similar levels. Pre-treatment with antagonists atropine and pirenzepine block the uptake of both proteins by about 80%. Conversely, AF-DX116 and PTX do not block uptake. **(D)** Left: Western blot analysis of cultured cells and neurons to analyze the presence of the M1 muscarinic receptor. Quantitation indicated that both HEK293 and neuronal cultures have a large amount of M1 muscarinic receptors compared to CHO cells. Right: Western blot analysis of HEK293 cells transfected with M1, M3, or M1/M3 muscarinic receptors to determine tau uptake. The introduction of the muscarinic receptors resulted in the significant uptake of tau into CHO cells. Scale bar: 20 μm. All experiments were performed three times with 10 images analyzed per experiment. (^∗^*p* < 0.05, ^∗∗^*p* < 0.01, ^∗∗∗^*p* < 0.001).

Propagation of abnormal tau in AD and other tauopathies has been proposed to occur through cell-to-cell transfer (as reviewed in Takeda, 2019). To better understand the mechanism of uptake, culture media spiked with recombinant proteins GST-tau, or GST-PH-Tau, 400 ng/mL (Supplementary Figure S1) was added to HEK293 or CHO cells and incubated for 4 h. After replacing the culture media, the cells were incubated in normal media for 2 days. Cells were washed to remove all extracellular tau, fixed and labeled with an anti-human tau antibody, counterstained with DAPI to observe the nuclei, and analyzed by confocal microscopy. Tau and PH-Tau were taken up into the HEK293 cells, but no uptake was observed in CHO cells (Figure 1A). GST alone was not detected in both cell lines (data not shown). These results indicate that tau, independent of phosphorylation state, can be uptaken in a cell-specific manner.

To determine if similar uptake can be observed in neuronal cells, primary mixed cerebellar neuronal cultures were prepared at Day 7 after birth (DIV1). Tau and PH-Tau, each at 400 ng/mL, were added to the media at DIV7 and incubated for 24 h. After incubation, the cultures were washed as above, fixed, processed for immunochemistry and double-labeled with anti-human tau and β-III tubulin (Figure 1B). DAPI was used as a counterstain to observe the nuclei. Both Tau and PH-Tau were taken up by neurons and accumulated at similar levels around the cell nucleus (Figure 1B, ROI). Uptake was observed without changes in neuritic morphology or neuronal numbers when comparing to control. Similar experiments were performed with human tau without a GST-tag and uptake was observed indicating that the uptake was due to tau and not the GST-tag (Supplementary Figure S2).

The different responses between HEK293 and CHO might be because HEK cells were generated from human embryonic kidney having properties similar to immature neurons and CHO cells are an epithelial cell line derived from ovaries of Chinese hamster (Shaw et al., 2002; Shafer and Williams, 2004; Lin et al., 2014). Similar to neurons, HEK293 cells express muscarinic receptors, while CHO cells do not. Neuronal muscarinic receptors have been shown to interact with tau (Gomez-Ramos et al., 2008; Gomez-Ramos et al., 2009; Avila et al., 2015). Western blot analysis of these cell lines shows that both HEK293 cells express about eight-fold higher M1 receptors (CHRM1) while primary cerebellar neuronal cultures express more than 20-fold higher M1 muscarinic receptors when compared to CHO cells (Figure 1D, left). Pre-treatment of cells with the muscarinic receptor antagonist atropine resulted in reduced uptake of both recombinant tau proteins in HEK293 cells and neuronal cultures (Figure 1A, bottom, Figure 1B, last column). Quantitative analysis shows that tau uptake in the neuronal cultures was reduced up to 80% of that without atropine (Figure 1C). Taken together these results implicate the involvement of muscarinic receptors in the uptake of tau into HEK293 cells and neurons.

### Muscarinic Receptor Subtypes M1 and M3 Are Involved in the Uptake of Tau

Given that tau is uptaken by HEK293 cells and neurons but not CHO cells, and uptake is blocked by atropine, we wanted to further analyze which of the five main subtypes of muscarinic receptors were involved. Primary cerebellar cultures were treated with pirenzepine, an M1 antagonist, AF-DX116, a selective M2 antagonist, and pertussis toxin (PTX), which blocks downstream processes of G_*i*_ G protein-coupled receptors (including M2/M4) (Figure 1C). Pirenzepine blocked the uptake of tau and PH-Tau about 80% which is similar to atropine. Conversely, both AF-DX116 and PTX did not block the uptake of either tau protein significantly. These results further implicate the M1 muscarinic receptor but not M2/M4 in the uptake of tau into the cells.

To further examine the role of muscarinic receptor subtypes in uptake into cells, CHO cells were transiently transfected with M1-HA, M3-HA, or both. The transfected cells were cultured for 48 h and then media containing the recombinant proteins was added as described above. The cells were collected after 24 h, washed, lysed and analyzed by immunoblot using anti-human tau. In CHO cells transfected with M1 or M3 there is ∼19-fold increase tau uptake (Figure 1D, right) compared to non-transfected CHO cells. When the cells were transfected with both M1 and M3 at the same time, the level of tau uptake increased by 31.5-fold. CHO cells transfected with either M1 or M3 appear to uptake tau at similar levels as HEK293 cells. When both receptors are present the uptake increases significantly. These results imply that both M1 and M3 are involved in the uptake of tau into cells.

### Consequences of Tau Exposure in Neuronal Processes

**FIGURE 2:**
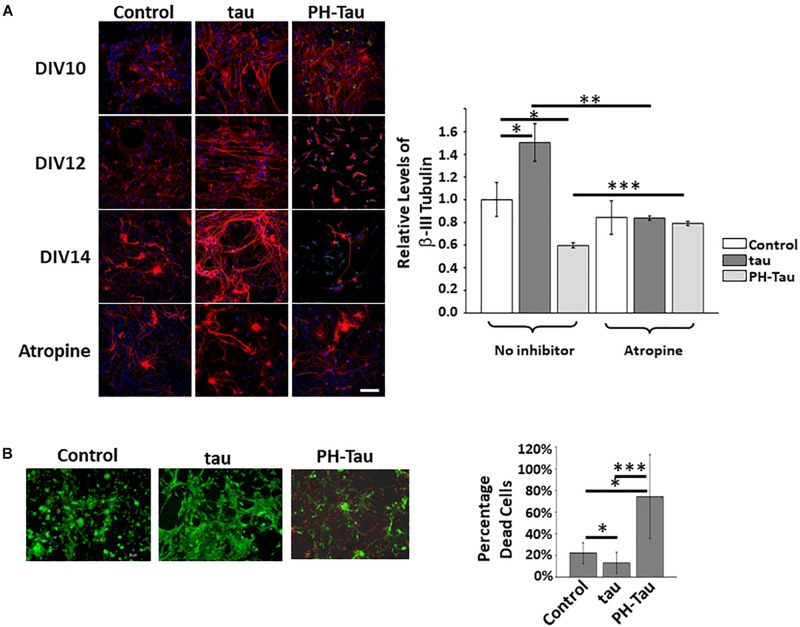
Uptake of tau affects the neuritic morphology of neurons in culture. **(A)** Proteins were added to the neuronal culture media as described. Over a period of 7 days, the neurons were fixed and studied by confocal microscopy. The addition of WT tau appears to stabilize and organize the neuronal processes whereas PH-Tau triggers a loss if neurites over time. Pre-treatment with atropine appears to block the uptake of tau and reduces changes in neuritic morphology. Graph: Quantitation of β-III tubulin indicates that tau (dark bars) increases protein levels whereas PH-Tau (light bars) decreases the levels indicating a loss of neuritic morphology. **(B)** LIVE/DEAD Imaging of neuronal cultures after treatment with tau or PH-Tau. The number of dead cells increases significantly in cultures treated with PH-Tau. Scale bar: 20 μm. All experiments were performed three times with 10 images analyzed per experiment. (^∗^*p* < 0.05, ^∗∗^*p* < 0.01, ^∗∗∗^*p* < 0.001).

To observe changes in neurons due to the uptake of tau or PH-Tau we performed the experiments as described above, where the neurons were exposed to tau for 24 h and cultures were allowed to grow and differentiate for up to seven more days *in vitro* (Figure 2A). βIII-tubulin intensity at DIV10 appears to be higher in all cultures to which tau was added when compared to the control cultures. After this point, the cultures treated with PH-Tau, showed decreasing intensity of βIII-tubulin. DIV12 neurons treated with PH-Tau began to lose neurites, a feature indicative of neurodegeneration. Interestingly, the few neurons in cultures treated with pathogenic tau, which are still present at DIV14, appear more similar to neuronal processes treated with wild-type tau than those in the control. Conversely, there was an increase in βIII-tubulin intensity in cultures treated with normal tau. DIV14 neurons treated with tau appear to bundle together and to be straighter than neurons in the control samples. Levels of βIII-tubulin in culture were quantitated at DIV14 based on the confocal acquired fluorescence (Figure 2A, graph). The quantitative analysis confirms that by DIV14, βIII-tubulin levels increased upon addition of tau and decreased when PH-Tau was added. Based on the observed changes in βIII-tubulin for each sample, it appears that the uptake of tau reinforces neuritic growth, while the uptake of PH-Tau triggers the loss of neurites and result in neurodegeneration similar to that observed in AD pathology.

As seen in Figure 1, the initial uptake of tau was blocked by atropine and when these cultures were allowed to incubate for 7 days, the neurons appeared similar to cultures without tau addition (Figure 2A, bottom images). Quantitative analysis of cultures at DIV14 of the exposed to tau with and without atropine pre-treatment show a significant decrease in βIII-tubulin intensity when atropine was present. The neudegeneration that was induced by exposure to PH-Tau was significantly inhibited by pre-treatment with atropine (Figure 2A, graph). These results indicate that muscarinic receptors play a role in the uptake of tau and that blocking them can control the uptake and therefore the overall condition of the neurons.

### Addition of PH-Tau Results in Increased Cell Death in Neuronal Cultures

To determine if this neuronal loss was due to cell death, neuronal cultures were prepared as described and were analyzed using the LIVE/DEAD Cell Imaging Invitrogen kit (Figure 2B). Live neurons stain green whereas cell death is indicated by red staining. There is a significant increase in the number of red cells in the neuronal cultures treated with PH-Tau when compared to the tau-treated and control cultures. Additionally, the cells (red or green) look mostly rounded, due to a loss of neuritic projections which is consistent with the results seen above when analyzing βIII-tubulin in treated cells. The percentage of dead cells in the neuronal cultures treated with wild-type tau is decreased 50% compared to control mixed primary neuronal cultures. This may be correlated to the increase in βIII-tubulin observed above. These results indicate that the addition of wild-type tau may play a role in strengthening the health of the neurons. Conversely, addition of PH-tau results in loss of neuritic projections and induction of cellular death which is similar to phenotypes seen in neurons of AD patients.

### Microglial Activation Is Triggered by the Presence of PH-Tau

**FIGURE 3:**
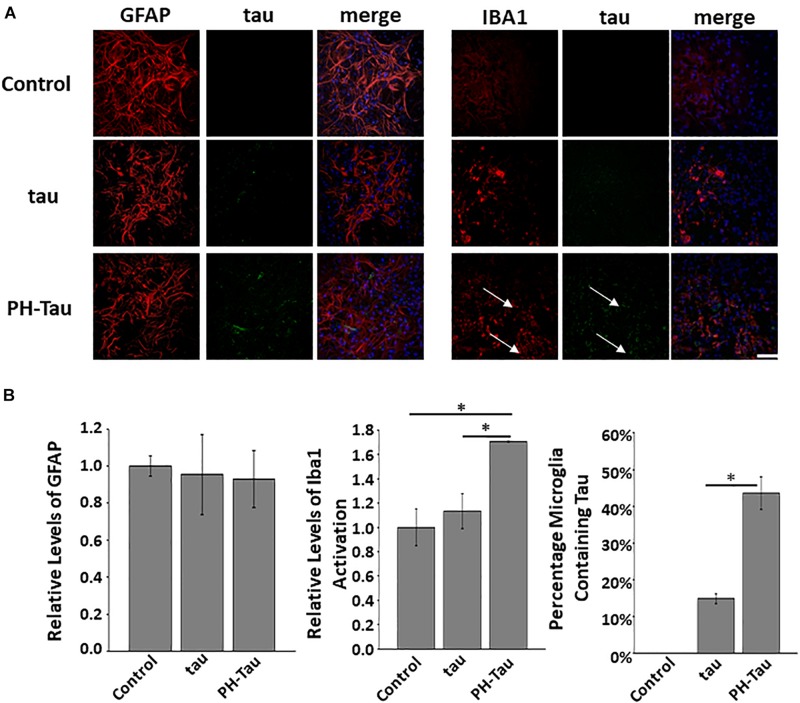
Uptake of tau affects the microglia but not astrocytes in neuronal culture. **(A)** Proteins were added to the neuronal culture media and analyzed after 7 days. There is no significant change in the amount of GFAP fluorescence in cultures challenged with either tau or PH-Tau when compared to control. The addition of the PH-Tau but not tau appears to activate the microglia which is correlated with the uptake of tau into the cells (white arrows). Scale Bar: 20 μm. **(B)** Left: Quantitation of the GFAP levels indicates that there is no change after addition of tau to cultures. Middle: Iba1 activation indicates that PH-Tau significantly activates the microglial response. Right: This activation correlates with the amount of tau uptaken by the microglia in culture. All experiments were performed three times with 10 images analyzed per experiment. (^∗^*p* < 0.05).

An important component of inflammation seen in brains of patients with AD other tauopathies is microglia activation. Therefore we investigated if in our experiments there was a microglia modification. The neuronal cultures analyzed contain a mixture of cells including neurons, microglia, and astrocytes (Supplementary Table S1). Primary cerebellar neuronal cultures were treated as described above and then washed and fixed at DIV14. Immunohistochemistry was performed with antibodies against GFAP, an astrocyte marker, and Iba1, a marker for activated microglia. It is important to note that other subpopulations of microglia may stain for Iba-1 (Ito et al., 1998), however, we are looking at relative changes upon addition of tau. No significant changes were found in the levels of astrocytes as judged by the levels of GFAP in the cultured neurons (Figure 3A, top). However, there was a significant increase in the activation of microglia in the cultures treated with PH-Tau when compared to control and tau-treated cultures (Figure 3A bottom, and Figure 3B left graph).

Further analysis of these cultures indicated that tau uptake into the microglia was dependent on the conformation of tau as PH-Tau was taken up to a much higher extent than the wild-type form (Figure 3B, right graph). The increase in microglial activation was directly correlated to the amount of tau uptake in the Iba1 active cells (Figure 3B, compare left and right graphs). In cultures treated with PH-Tau, 43% of activated microglia were also positive for human tau. Cultures treated with tau had significantly lower proportion of tau containing microglia which correlates to an insignificant level of Iba1 activation (Figure 3B, graphs). An increased microglial response could be indicative of conditions that may lead to inflammation, similar to that found in patients with AD.

### PH-Tau Effectively Mimics AD P-Tau Taken From Human Brain Tissue

**FIGURE 4:**
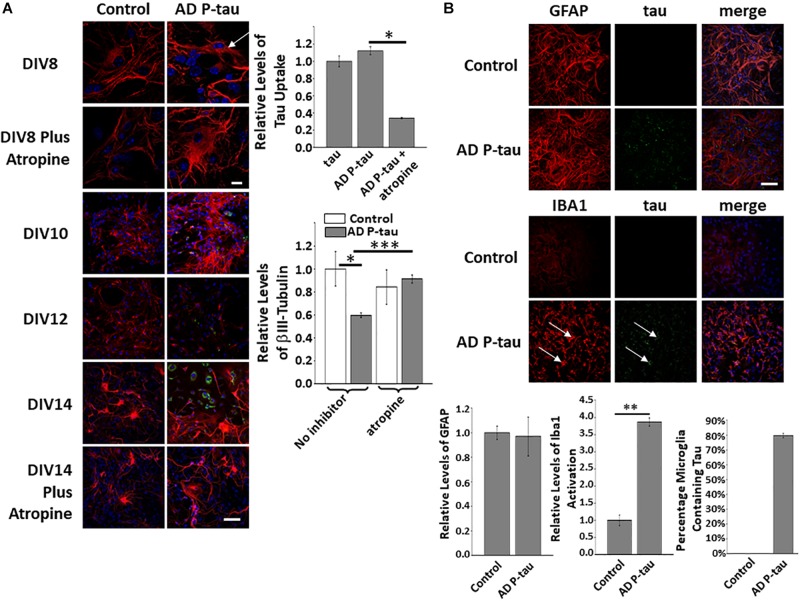
AD P-tau addition to neuronal cultures has similar effects as PH-Tau. **(A)** AD P-tau is uptaken after incubation of 24 h and atropine blocks this uptake. The white arrow indicates where tau (green) is localized near the nucleus. No tau is observed in neurons pre-treated with atropine. Quantitation of uptake is observed in the top graph on the right. At DIV10, β-III tubulin appears to increase compared to control followed by a sharp decrease over time, see DIV12 and DIV14. The pre-treatment with atropine blocks the decrease in β-III tubulin levels. These changes are observed by confocal microscopy (images) and by quantitation of tau uptake (bar graph, top right) or β-III tubulin fluorescence (bar graph, bottom right). **(B)** Activation of microglia is observed upon addition of AD P-tau and this activation is correlated with the uptake of protein (indicated by the white arrows). GFAP levels are observed in the top row and activation of microglia is observed by an increase in fluorescence of Iba1. Bar graphs show levels of GFAP fluorescence (left), quantiation of Iba1 activation (middle) and AD P-tau uptake (right) Scale bar: 20 μm. All experiments were performed three times with 10 images analyzed per experiment. (^∗^*p* < 0.05, ^∗∗^*p* < 0.01, ^∗∗∗^*p* < 0.001).

Alzheimer’s disease P-tau, soluble, hyperphosphorylated tau extracted from the brains of human patients was used in similar experiments to those described above. The uptake of AD P-tau was shown to be similar to tau and PH-Tau and is also blocked by atropine (Figure 4A top and top graph). Cultures treated with PH-Tau followed a similar pattern of neurodegeneration compared to the addition of AD P-tau, showing increased βIII-tubulin at DIV10 followed by a decrease compared to control (Figure 4A bottom and bottom graph). Furthermore, pre-treatment with atropine resulted in very little loss of βIII-tubulin compared to control. Finally, in cultures treated with AD P-tau, no change was observed in GFAP staining, but microglia were activated almost four-fold more than control cells and almost 80% of these activated microglia were also positive for human tau. Taken together, these results confirm the validity of PH-Tau as a good mimic for AD P-tau and can be used as a surrogate in future studies.

### Histological Evaluation of Neurodegeneration in CA3 Region of the Hippocampus

**FIGURE 5:**
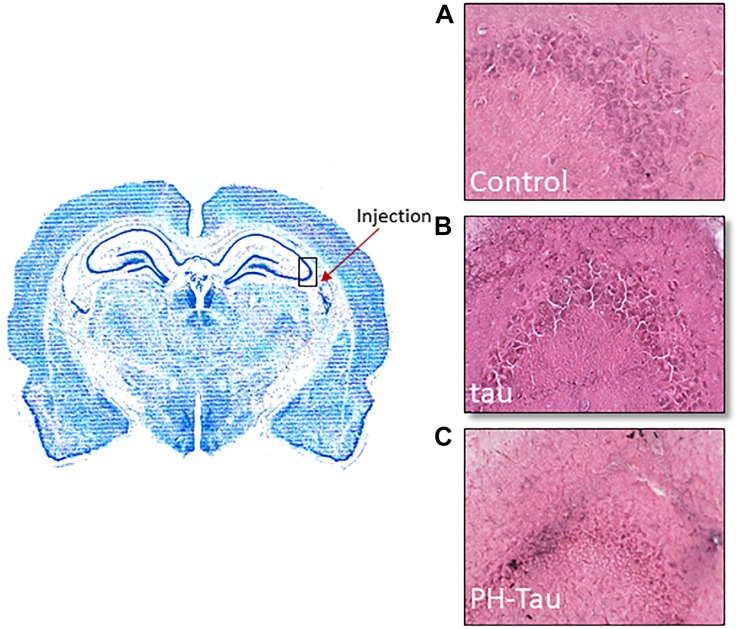
Neurodegeneration observed in the CA3 region of the hippocampus. Stereotaxic intracranial injections were performed using **(A)** aCSF, **(B)** tau, and **(C)** PH-Tau. After 45 days, the brains were harvested and prepared by cryosectioning after fixation. The arrow indicates the injection site and the box represent the imaged area shown on the right panel. H&E staining was performed to follow changes in nerons in the CA3 region. Healthy neurons were observed in the aCSF and wild-type tau injections whereas condensed nuclei and changes in cellular morphology was observed when PH-Tau was injected.

Mice received a stereotaxic intracranial injection of pure artificial cerebral spinal fluid (aCSF) or either tau or PH-Tau dissolved in aCSF. Forty-five days post injection, mice were perfused with 4% paraformaldehyde and brains were dissected and post fixed for 2 h. Coronal cryosections were stained with Haemotoxylin and Eosin (H&E). These images are representative of the (A) aCSF-injected, (B) tau-injected, and (C) PH-Tau-injected mice (Figure 5). The arrow indicates the injection site and the box represents the imaged area shown on the right panels. The aCSF and wild-type tau injected mice show relatively healthy neurons as demonstrated by the light appearance of neurons and their morphological integrity. Whereas the PH-Tau injected mice show relatively unhealthy neurons as evident from nuclear condensation based on intense staining and the irregular shape of the cell body.

## Discussion

In this study, we have observed that recombinant tau and pathological tau can be taken up from the culture media into cell lines, microglia, and neurons and that this uptake activity involves muscarinic receptors. The addition of normal tau to neurons promotes neuritic extension, while PH-Tau leads to the disruption of neuritic processes and accumulation in the somatodendritic compartment. These findings suggest a novel mechanism for the pathogenesis of hyperphosphorylated tau and demonstrate additional functions for normal tau. We have further confirmed our previous study which showed that PH-Tau mimics AD P-Tau in cultured neurons. Both forms of tau caused the disruption of neuritic processes and accumulation of tau aggregates in the somatodendritic compartment.

The addition of tau to cell and neuronal cultures has been previously studied (Frost et al., 2009; Nonaka et al., 2010; Guo and Lee, 2011; Wu et al., 2013; Sanders et al., 2014; Wu et al., 2016; Evans et al., 2018). Research has indicated that the minimal unit for uptake is trimeric tau (Mirbaha et al., 2015). These tau trimers also appear to be the minimal tau toxic unit in both AD and PSP (Shafiei et al., 2017). Native gel electrophoresis indicates that the tau and PH-Tau that we are using are both mixtures of oligomeric states but that PH-Tau appears to be composed of a greater number of higher order complexes (Supplementary Figure S3). Furthermore, when seeded with AD P-tau the number of higher oligomeric complexes increases indicating the seeding ability of pathological tau. Our experiments show that normal tau is taken up similarly to PH-Tau. However, only PH-Tau exhibited a toxic effect indicating that it may not be the size of the aggregate that is toxic, but the phosphorylation state of tau.

To determine the importance of muscarinic receptors in intracellular uptake of tau, we added normal tau or PH-Tau to the culture media of HEK293 cells or CHO cells. Uptake of tau was only observed in HEK293 cells. Unlike HEK293 cells, CHO cells do not naturally express muscarinic receptors. Transfection of CHO cells with either M1, M3, or M1/M3 muscarinic receptors indicated that the presence of these receptors was necessary for tau uptake (Figure 1D). To further investigate the role of muscarinic receptors, cell cultures were pre-treated with atropine, a known muscarinic receptor antagonist, prior to the addition of tau. Atropine blocked tau uptake in HEK293 cells, indicating that the presence of muscarinic receptors is correlated with tau uptake. It is known that cortical neurons, including hippocampal cells have M1 and M3 muscarinic receptors and so do glial cells (Levey et al., 1994). Neuronal cultures were used to determine if both forms of tau could be uptaken in the brain. Tau, PH-Tau, and AD P-tau were all uptaken at similar levels and this uptake was blocked up to 80% by atropine (Figures 1B,C, 4). The M1 anatagonist pirenzepine blocked uptake in a similar fashion as atropine, but AF-DX116 and pertussis toxin, were both unable to block uptake. These results implicate M1 receptors in the uptake of tau (Figure 1C). Interestingly, a cholinesterase inhibitor is one of the medications prescribed to ameliorate some of the cognitive deficits of AD (Birks, 2006; Soukup et al., 2017). The present findings that atropine blocked the PH-Tau uptake could provide an explanation for the limited efficacy of this therapeutic intervention. Acetylcholine is one of the transmitters in the cortical and hippocampal circuits, and because PH-Tau transfer to other neurons may be blocked, other, non-effected neurons, could maintain the synaptic transmission prolonging persistence of acetylcholine in the synaptic cleft. Complementary to this cholinergic mechanism was the study of Schmitz et al. (2016), patients with early stages of AD, clinically silent, have abnormal degeneration of cholinergic neurons.

From our experimental evidence, neurons treated with wild-type tau that were cultured until DIV14 were more organized and had many more aligned neuritic processes compared to the untreated neurons (Figure 2). These findings suggest that extra-neuronal tau might be a physiological signal. Although the presence of tau in the extracellular space is described, there is no consensus about physiological function for this localization of tau (Chai et al., 2012; Magnoni et al., 2012; Karch et al., 2013; Bright et al., 2015; Kanmert et al., 2015). Tau increases the electrical activity of iPSC-derived or primary cortical neurons, in line with the finding that tau leads to intracellular calcium increase (Gomez-Ramos et al., 2008; Bright et al., 2015). As such, extracellular tau may play an important neuromodulatory role for cognition. In agreement with our results, Biundo et al. (2018) have shown that tau depletion in a mouse model led to age-dependent deficits in memory and synaptic plasticity, correlating with levels of normal tau expression. Considering the potential beneficial role of normal tau with respect to the electrophysiological functioning and morphology of neurons, our study further explored the effects of pathological tau. Addition of PH-Tau or AD P-tau to the culture medium induced a retraction of the neuritic processes (Figures 2, 4). This retraction by PH-Tau is noticeable 5 days (DIV12) after exposure (Figure 2). Similarly, at 45 days post injection of mice injected with PH-Tau, the neuronal nuclei began to condense and the cell bodies did not appear rounded (Figure 5). Though uptake of wild-type tau and PH-Tau both appear to be mediated via similar muscarinic receptors, the effects on neurons and microglia are opposite in nature. The downstream effects of each of these proteins should be further investigated.

Microglia activation, as indicated by an increase in Iba1 staining in cultures, after treatment with PH-Tau and ADP-Tau, while astrocytes were left unchanged (Figures 3, 4). This may indicate a response to the presence of pathological tau. Microglia are capable of taking up extracellular tau and have the ability to degrade the protein to remove it from the matrix (Bolós et al., 2016). Murine microglial cells from young mice have been used to clear tau from brain slices of P301S mice and from AD patients (Luo et al., 2015). Other studies have shown that microglia cluster in tau-rich regions indicating that they may play a role in the clearance of pathogenic tau (Bolós et al., 2016; Davies et al., 2017). Interestingly, there is a report of an increase in microglia that express muscarinic receptors, in particular M3, in AD mouse models (Pannell et al., 2016). Tau has been shown to bind to M1 and M3 mucarinic receptors (Gomez-Ramos et al., 2008). Therefore, activation of microglial cells that express M1 and M3 muscarinic receptors in the presence of extracellular tau for clearance further supports our findings that these specific receptor subtypes are important for tau uptake.

**FIGURE 6:**
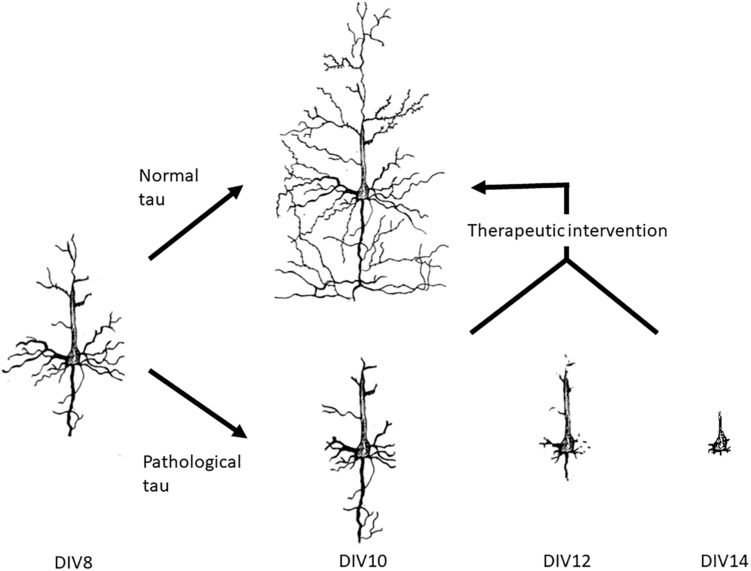
Neuronal culture model system for the development of therapeutic interventions. We have shown that the neuronal culture system that we use can mimic neurodegeneration within 2 weeks of preparation. At DIV7, tau proteins are added to the culture medium for 24 h before exchanging the media to tau free media. Uptake of the proteins can be observed at DIV8. By DIV10, no tau is observed in the normal tau cultures, but the levels of βIII-tubulin increase. Cultures treated with pathogenic tau began to degenerate by DIV10 with significant loss of βIII-tubulin by DIV14. This system can be used to try out novel therapeutics to either halt degeneration and/or return the neurons to their original form.

In this report, novel function and pathophysiology of tau in the extracellular space were investigated and neurons treated with pathological tau mimic the neurodegeneration observed in AD (Figure 6). This versatile model allows for the potential development of high throughput screening of novel therapeutics against tau-induced neurodegeneration. We showed that the uptake of tau is correlated with microtubule stabilization, and that the uptake of wild-type tau can increase stabilization and organization of microtubules whereas uptake of PH-Tau causes destabilization leading to neurite retraction. With these neuronal cultures therapeutic interventions can be tried at varying points of neurodegeneration with the hope of allowing the neurites to regenerate over time. Further analysis is necessary to understand the mechanisms of tau propagation, nucleation of oligomerization and induction of neurodegeneration.

With the present findings we hypothesize that under normal physiological conditions, tau is released in the extracellular space, uptaken by other neuronal cells in a muscarinic-receptor mediated pathway, and the connectivity of the neurons is reinforced. Early onset of AD progression begins with the appearance of pathological tau in a subset of neurons that can be secreted in the microenvironment, spreading its toxicity to the neighboring cells. Tau can be released as a naked pathological tau aggregates or in vesicles or exosomes. Pathological tau uptake by microglial cells induces activation, initiating an inflammatory response. Pathological tau exhibits a prion-like behavior inducing conformational changes in normal tau, disrupting microtubules or through other pathways, causing neurodegeneration. This sequence of events suggests a mechanism of propagation that exacerbates pathological conditions and that it could be a target for therapeutic intervention.

## Data Availability

The datasets generated for this study are available on request to the corresponding author.

## Ethics Statement

Animal Subjects: The animal study was reviewed and approved by the College of Staten Island (CSI) Office for the Protection of Research subjects *via* the Institutional Animal Care and Use Committee.

## Author Contributions

All authors contributed in the critical discussion of the experimental designs, results, and writing of the manuscript. VM performed the protein purifications, cell and primary neuronal culture, and immunocytochemistry and confocal imaging. LC performed the native gel electrophoresis and participated in the recombinant protein purification. AE-HM, AS, and AEL participated in the *in vivo* recombinant tau and PH-Tau stereotactic injection. GP participated in the design of the pharmacological inhibition of muscarinic receptors experiments. AA supervised the research project.

## Conflict of Interest Statement

The authors declare that the research was conducted in the absence of any commercial or financial relationships that could be construed as a potential conflict of interest.
