# Supplementary material for: Normal and Pathological Tau Uptake Mediated by M1/M3 Muscarinic Receptors Promotes Opposite Neuronal Changes
Source: Front Cell Neurosci. 2019 Sep 4;13:403. doi: 10.3389/fncel.2019.00403 (PMC6737038; doi:10.3389/fncel.2019.00403)
Supplement: Supplementary file 1 [file Data_Sheet_1.pdf]

Supplementary Material

Normal & Pathological Tau Uptake Mediated by M1/M3 Muscarinic Receptors Promotes Opposite Neuronal Changes

Viktoriya Morozova [1,2], Leah S. Cohen[1], Ali El-Hadi Makki[1], Alison Shur[1,2], Guillermo Pilar[3], Abdeslem ElIdrissi[1,2], Alejandra D. Alonso[1,2]*

[1]Department of Biology and Center for Developmental Neuroscience, College of Staten Island, The City University of New York, Staten Island, New York 10314, USA.

[2]Biology Program, The Graduate Center, The City University of New York, New York, New York 10016, USA.

[3]Guillermo Pilar, Department of Neurosciences,Case.Western Reserve University. Cleveland OH 44106 USA

*** Correspondence:**
Alejandra D. Alonso
Alejandra.Alonso@csi.cuny.edu

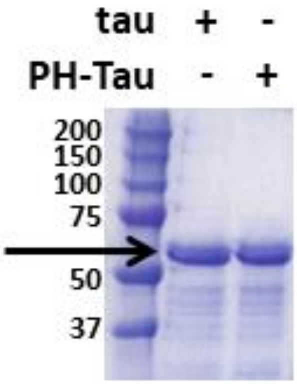

Supplemental Fig. S1. SDS-PAGE analysis of GST-tau and GST-PH-Tau. GST-tau and GST-PH-Tau were heterologously expressed as described. The proteins were analyzed by SDS-PAGE for purity. Prior to loading the proteins were quantitated using the Bradford Assay. These proteins were used in the experiments described in this research study.

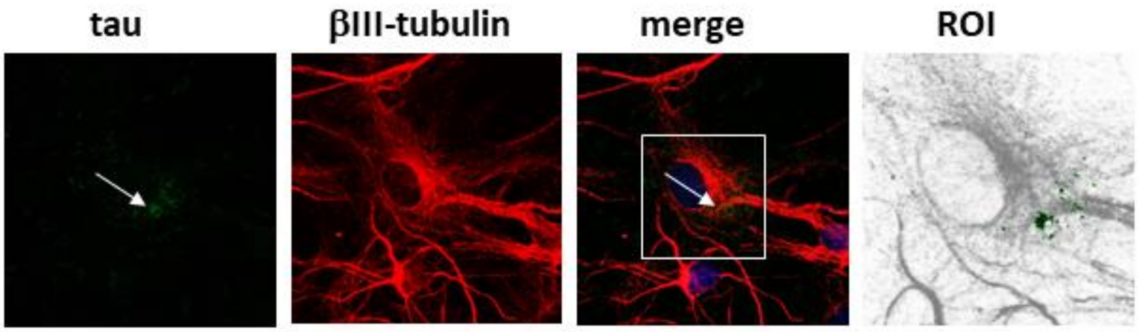

Supplementary Fig. S2. Uptake of tau without fusion protein. The tau protein without a fusion tag was heterologously expressed and used uptake assay in neuronal cultures. Tau was uptaken in a similar fashion as the fusion proteins indicating that the uptake was tau-dependent and not due to the presence of the GST-tag.

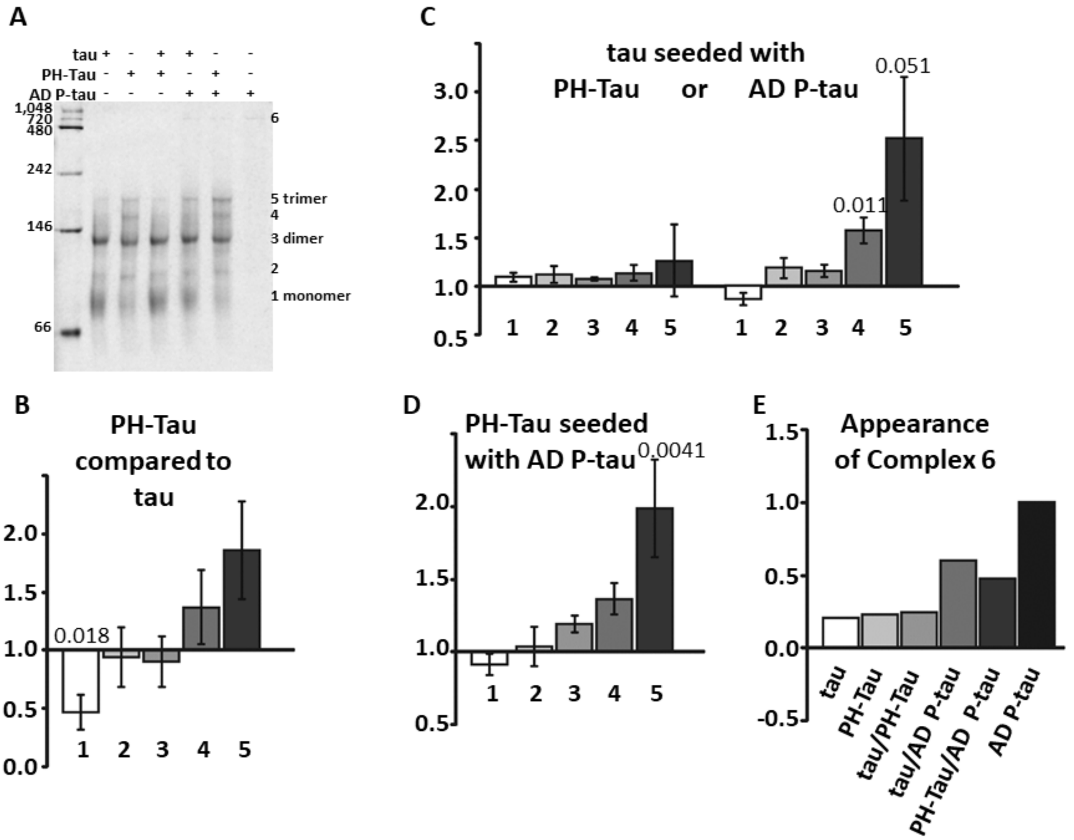

Supplemental Fig. S3. Oligomeric stoichiometry of tau changes depending on phosphorylation state or seeding. (A) Native PAGE analysis of GST-tau and GST-PH-Tau were analyzed by Native gel electrophoresis on a 10% gel with Native sample buffer (62.5 mM Tris-HCl, pH 6.8, 25% glycerol, 1% bromophenol blue) without sonication prior to loading, except for the AD P-tau which was sonicated in a cup sonicator at room temperature for 1 hour at 50W.The oligomeric stoichiometry appears to be different when comparing the wild-type tau to the pseudophosphorylated form. Furthermore, upon addition of substoichiometric amounts of AD P-tau or PH-Tau, the GST-tau appears to change its oligomeric stoichiometry. **(B)** Changes in oligomeric states by the addition of pseudophosphorylation sites were observed when comparing GST-PH-Tau to GST-tau. These changes are mainly with the loss of monomer and the increase of trimer. **(C)** GST-tau was seeded with GST-PH-Tau (left) and AD P-tau (right) at a 25:1 mole ration (GST-tau to seeding protein). The increase in the formation of the trimer was more prevalent in the AD P-tau seeding than the GST-PH-Tau. **(D)** Seeding by AD P-tau of GST-PH-Tau resulted in an even further increase in the presence of the trimer when comparing the seeding reaction to the GST-PH-Tau alone. **(E)** The appearance of complex 6 in the reactions seeded by AD P-tau was faint but highly reproducible.

As we have described in the accompanying paper, PH-Tau and AD P-tau addition to neuronal cultures results in similar process disruption, and suggests that PH-Tau can mimic of AD P-tau. We hypothesize that tau modifications change its conformation and/or affect its ability to form oligomers. In an attempt to characterize the changes in tau conformation and/or oligomerization, we analyzed tau and PH-Tau using native polyacrylamide gel electrophoresis (Supplemental Fig. S3). Several different bands could be detected and by comparison to the Native Mark molecular weight standards (Invitrogen) we could identify a monomeric (band 1), a dimeric (band 3), or a trimeric (band 5) oligomeric state. Other bands

were observed, one high molecular weight aggregate (band 6) and an intermediate state between monomeric and dimeric forms (band 2) and one between dimeric and trimeric forms (band 4). Though both tau and PH-Tau had monomers and dimers, clear differences in their oligomeric stoichiometry could be observed (Supplemental Fig. S3B). The levels of monomer in the PH-Tau were decreased 0.5-fold when compared to tau (p=0.018). Conversely, there was an increase of 1.9-fold in the trimer conformation of PH-Tau.

Observed experimental changes in the oligomeric stoichiometry may be indicative of nucleation of aggregation, *in vivo*, by pathological forms of tau. To confirm a "prion-like behavior" of pathological forms of tau, 1 mole of PH-Tau or AD P-tau was mixed with 25 moles of tau, incubated at room temperature for 30 minutes, and then analyzed using native polyacrylamide gel electrophoresis (Supplemental Fig. S3A, S3C, and S3E). PH-Tau caused a 1.3-fold increase in the trimeric conformation. More strikingly, the addition of AD P-tau to the tau protein resulted in significant increases in the trimeric conformation (2.5-fold, p=0.051) and the intermediate conformation band 4 (1.6-fold, p=0.011). With this mixture, the pattern resembles more the pattern of PH-Tau than that of tau (compare lanes 1, 2 and 4, Supplemental Fig. S3A, and graphs Supplemental Fig. S3B and C). The appearance of the high molecular weight aggregate (band 6), absent from the normal tau cannot be statistically quantitated as the intensity of the band is low, but its appearance is reproducible over multiple experimental trials (Supplemental Fig. S3E). This change in oligomeric stoichiometry of tau, which is nucleated by AD P-tau, confirms the prion-like activity of AD P-tau consistent with our previously published results (Alonso, Grundke-Iqbal et al. 1996). Comparison of PH-Tau and the pronounced effect of AD P-tau nucleation of tau oligomerization compared to PH-Tau, which added in similar stoichiometry, indicated PH-Tau is less potent in transferring its oligomeric state to the wild-type protein (Supplemental Fig. S3C, left). Addition of AD P-tau to PH-Tau induced an increase in the proportion of the trimer conformation (2.0-fold, p=0.0041, Supplemental Fig. S3D) and the high molecular weight aggregates (Supplemental Fig. S3E).

Cell type	%	SD
Neurons	31.25	8.13
Astrocytes	42.75	1.06
Activated microglia	19.65	5.16

Supplemental Table 1. Composition of cell types in neuronal cultures.

References:

Alonso, A. D., I. Grundke-Iqbal and K. Iqbal (1996). "Alzheimer's disease hyperphosphorylated tau sequesters normal tau into tangles of filaments and disassembles microtubules." Nat Med **2**(7): 783-787.
